# Enhanced removal of heavy metal ions from aqueous solution using manganese dioxide-loaded biochar: Behavior and mechanism

**DOI:** 10.1038/s41598-020-63000-z

**Published:** 2020-04-08

**Authors:** Haipeng Zhang, Fangfang Xu, Jinyuan Xue, Shiyong Chen, Juanjuan Wang, Yanju Yang

**Affiliations:** 1 Jiangsu Key Laboratory of Crop Genetics and Physiology, Jiangsu Co-Innovation Center for Modern Production Technology of Grain Crops, Yangzhou University, Yangzhou, 225009 China; 2 College of Environmental Science and Engineering, Yangzhou University, Yangzhou, 225009 China

**Keywords:** Environmental chemistry, Chemical engineering

## Abstract

In this study, a redox precipitation method was used to load manganese dioxide (MnO_2_) nanoparticles on biochar (BC) (BC@MnO_2_) pyrolyzed from the invasive water hyacinth, and the adsorption of Cd(II),Cu(II), Zn(II), and Pb(II) was investigated. Several techniques were used to characterize the adsorbents. The results revealed that the BC surface was covered by many intertwined thin amorphous MnO_2_ nanosheets, which significantly increased its specific surface area and pore volume. The adsorption of heavy metal ions by BC was negligible, whereas the MnO_2_-containing adsorbents exhibited a high capacity for adsorbing heavy metal ions. However, the MnO_2_-normalized adsorption amount decreased with increasing MnO_2_ load and was largely unchanged at MnO_2_ loads of 26.6% to 30.2%. The capacity for adsorbing heavy metal ions of BC@MnO_2_ was pH-dependent, but the adsorption affinity was unaffected by coexisting ions. Column tests revealed that BC@MnO_2_ with a load of 26.6% had a high capacity for removing heavy metal ions from simulated and real electroplating wastewater. Therefore, BC@MnO_2_ with a load of 26.6% shows promise as a regenerable adsorbent for removing heavy metal ions from water/wastewater. This study could lay an essential foundation to develop a win-win strategy for heavy metal ions removal from wastewater using biochar derived from water hyacinth.

## Introduction

Increasing industrial wastewater pollution has become a global concern, and wastewater often contains dyes, heavy metal ions, phosphates, arsenic, or other toxic and non-biodegradable substances^1^. Industrial wastewater must be disposed of prior to discharge to avoid contamination of the water supply. Many countries have enacted strict laws to suppress the discharge of heavy metal ions in industrial wastewater^2^. Precipitation, adsorption, biological treatment, and other methods are used to remove heavy metal ions from industrial wastewater^3^. Adsorption is a widely used and effective method^4^, and developing adsorbents for removing heavy metal ions is a research priority.

Nanosized metal oxides (*e.g*., zirconium oxides^5^, iron oxides^6^, aluminum oxides^7^, and manganese oxides^8^) are potential adsorbents because of their large surface areas, abundance of defect sites, and high surface to bulk atom ratios. Compared with their bulk counterparts, nanosized metal oxides exhibit better performance for adsorbing heavy metal ions^4,8^. Manganese oxides are excellent adsorbents because of their ability to form complexes with heavy metal ions (*e.g*., Cd(II), Cu(II), Zn(II), and Pb(II)) and good chemical stability under basic and acidic conditions^9^. For instance, Al Degs *et al*. reported that nanosized manganese dioxide (MnO_2_) exhibited greater adsorption of lead ions within a wide pH range^10^. Zhang *et al*. concluded that nanosized manganese oxide demonstrated high adsorption affinity for Pb(II), Cd(II) and Cu(II) ions in aqueous solution and was not significantly affected by coexisting ions (Na^+^ and Mg^2+^)^9^. Wan *et al*. reported that adsorption of heavy metal ions by layered MnO_2_ nanoparticles was dependent on complexation with Mn-OH groups on the surface^11^. Notably, only nanosized MnO_2_ has an active surface area^12,13^ but it is likely to form aggregates because of its high surface energy^14^, which can greatly reduce the specific surface area and significantly decrease the capacity for adsorbing heavy metal ions. Nanosized MnO_2_ can be supported on a carrier with a large surface area to enhance its dispersibility and adsorption. Nanosized MnO_2_ loaded on materials with a high specific surface area—such as graphene oxide^11^, ordered mesoporous silica^15^, and ordered mesoporous carbon materials^16^ exhibited higher performance in energy storage, catalysis, and adsorption than aggregated nanosized MnO_2_. However, materials with large specific surface areas are costly and difficult to manufacture, which restricts their large-scale preparation and application. Hence, the loading of nanosized MnO_2_ onto low-cost supporting materials with large specific surface areas shows promise.

Biochar (BC) is produced via pyrolysis of organic feedstocks at <700 °C under oxygen-limited conditions. Unfortunately, blank BC often exhibits relatively low adsorption efficiency for heavy metal ions^4,17^. However, due to its abundant surface functional groups, availability, and low cost, BC is suitable for hosting metal oxides for adsorption and catalysis applications^7^. For example, metal hydroxides such as Fe-Mn binary oxides^18^, iron oxides^19,20^, aluminum oxides^21,22^, and silicon^23^ have been introduced to the inner and outer surfaces of BC. Sun *et al*.^24^, Qiu *et al*.^25^, and Li *et al*.^26^ studied the application of MnO_2_-loaded BC for removing Pb(II), Cd(II), and fluoroquinolone antibiotics, respectively. Prior studies have focused on the preparation and adsorption performance of MnO_2_-loaded BC. However, the means of enhancing MnO_2_ loading and reactions among BC, MnO_2_, and target contaminants have been overlooked.

The objectives of this study were to explore (1) the effect of loading different mass ratios of MnO_2_ on the removal of heavy metal ions by BC supported MnO_2_, (2) the potential of this adsorbent material for removing heavy metal ions, and (3) the mechanisms of adsorption of heavy metal ions. The adsorbents were prepared using potassium permanganate as a precursor and by loading MnO_2_ onto BC using the redox precipitation method. X-ray diffraction (XRD), X-ray photoelectron spectroscopy (XPS), transmission electron microscopy (TEM), N_2_ adsorption-desorption assay, and measurement of zeta potential were used to characterize the adsorbents. The adsorption of heavy metal ions (Pb(II), Cd(II), Cu(II), and Zn(II)) by the adsorbents was examined using batch experiments and column tests.

## Results and discussion

### Adsorbent characteristics

The X-ray fluorescence results (Table 1) revealed that the MnO_2_ load in the BC@MnO_2_ was 12.3%, 18.4%, 26.6%, and 30.2% respectively, and indicating that MnO_2_ was successfully loaded onto the BC surface. The Mn(IV) contents in BC@MnO_2_ were further determined, and the results are shown in Table 1. The Mn(IV) contents in BC@MnO_2_-12.3, BC@MnO_2_-18.4, BC@MnO_2_-26.6 and BC@MnO_2_-30.2 were 6.9%, 10.7%, 16.0% and 17.8%, respectively.

**Table 1:** Structural properties of the adsorbents.

Sorbents	MnO_2_ content^a^ (wt.%)	Mn(IV) content^b^ (wt.%)	*S*_BET_ (m^2^ g^−1^)	*V*_p_^c^ (cm^3^ g^−1^)	*d*_p_^d^ (nm)
BC	0.6	0.5	3.5	0.01	4.1
BC@MnO_2_-12.3	12.3	6.9	135.9	0.19	3.7
BC@MnO_2_-18.4	18.4	10.7	181.9	0.25	3.7
BC@MnO_2_-26.3	26.6	16.0	120.2	0.14	3.5
BC@MnO_2_-30.2	30.2	17.8	12.5	0.03	2.0

^a^Determined by X-ray fluorescence.

^b^Determined by using oxalic acid-permanganate back-titration method.

^c^Total pore volume, determined at *P*/*P*_0_ = 0.97.

^d^Most probable pore diameter, determined by BJH pore size distribution.

**Figure 1:**
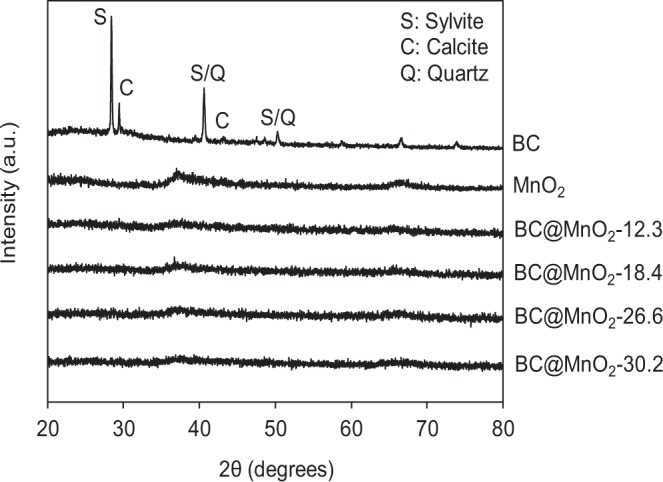
X-ray diffraction spectroscopy (XRD) of pristine BC and BC@MnO_2_ composites (S = sylvite, C = calcite, Q = quartz).

XRD analysis (in the 2*θ* range 20–80°) was performed to characterize the crystalline structures of BC and BC@MnO_2_ (Fig. 1). For BC, the diffraction peaks at 28.4°, 29.4°, 40.6°, 43.4°, and 50.4° were assigned to sylvite, calcite, and quartz, respectively. These peaks agree with previously reported diffraction patterns^2,27,28^. After MnO_2_ deposition, BC@MnO_2_ exhibited a broader and lower intensity peak at 37.4°, which is characteristic of amorphous MnO_2_^9^. No additional peaks were detected in the diffraction patterns of BC@MnO_2_ adsorbents, suggesting that no secondary products or unreacted input compounds were loaded during the synthesis of BC@MnO_2_.

**Figure 2:**
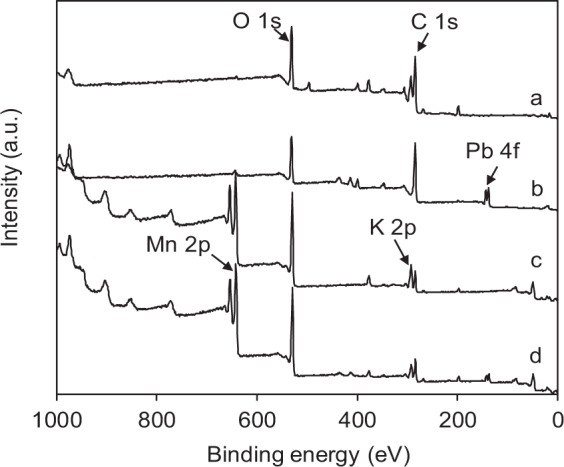
Full-scan XPS spectra of (**a**) BC, (**b**) BC after Pb(II) adsorption, (**c**) BC@MnO_2_-26.6, (**d**) BC@MnO_2_-26.6 after Pb(II) adsorption.

XPS analysis was used to verify the loading of MnO_2_ on BC. The XPS spectra of BC and BC@MnO_2_-26.6 are presented in Fig. 2. For BC, a C1s peak was present at 284.6 eV and Mn 2p peaks were detected for BC@MnO_2_-26.6, confirming the loading of MnO_2_ on BC.

**Figure 3:**
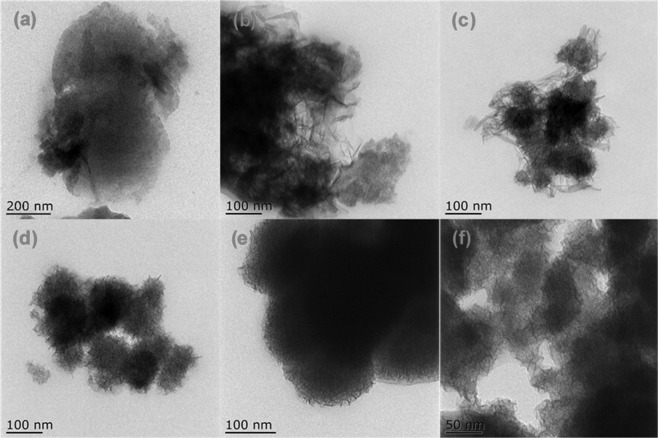
TEM images of (**a**) BC, (**b**) BC@MnO_2_-12.3, (**c**) BC@MnO_2_-18.4, (**d**) BC@MnO_2_-26.6, (**e**,**f**) BC@MnO_2_-30.2.

TEM was conducted to visualize the surface morphology of BC and BC@MnO_2_ (Fig. 3). In contrast to the surface of BC, that of BC@MnO_2_ exhibited many structures with a flossy or fluffy pattern. According to the XRD results, the multi-branch hierarchical nanostructures of MnO_2_ consisted of many intertwined thin amorphous MnO_2_ nanosheets on the BC surface. These three-dimensional hierarchical microspheres likely provided abundant adsorption sites and so enhanced the adsorption of heavy metal ions from aqueous solution.

The N_2_ adsorption-desorption isotherms (Fig. S1a) and pore size distributions of the samples at 77 K (Fig. S1b) verified the above results. The Brunauer–Emmett–Teller (BET) surface areas and pore volumes are listed in Table 1. Due to its large particle size, BC had a small BET surface area (3.5 m^2^ g^−1^), but its BET surface area increased significantly when MnO_2_ was deposited. MnO_2_-loaded BC with varied MnO_2_ loading amounts exhibited higher specific surface areas than BC, reflecting the increase of specific surface area of BC after MnO_2_ modification. The specific surface area of BC@MnO_2_ increased from 135.9 m^2^ g^−1^ to 181.5 m^2^ g^−1^ when MnO_2_ loading amount increased from 12.3% to 18.4%. However, the specific surface area of BC@MnO_2_ began to decrease with the further increase of MnO_2_ loading amount. The initial increase may be explained by the loading of MnO_2_ nanoparticles and generation of more micropores, while the decrease at high loading is likely due to excess deposition of MnO_2_ nanoparticles, which may lead to the pores blockage and destruction of some micropore structure^17,20^. In parallel, with the increase of MnO_2_ loading amount, total pore volume (*V*p) increased firstly, then decreased with the further increase of MnO_2_ loading amount. This is possibly owing to the highly excessive KMnO_4_ dosage leading to high burn-off level of the inner structure of biochar, resulting in the formation of mesopores due to collapse and growth of existing micropores structure^24,25^. The pore diameter of BC became lower after MnO_2_ modification. The decrease of pore diameter might be explained by the deposition of MnO_2_ nanoparticles leading to the pores blockage. With the increase of MnO_2_ loading amount from 12.3% to 30.2%, the pore size decreased from 3.7 nm to 2.0 nm, confirming the existence of pore-blocking.

**Figure 4:**
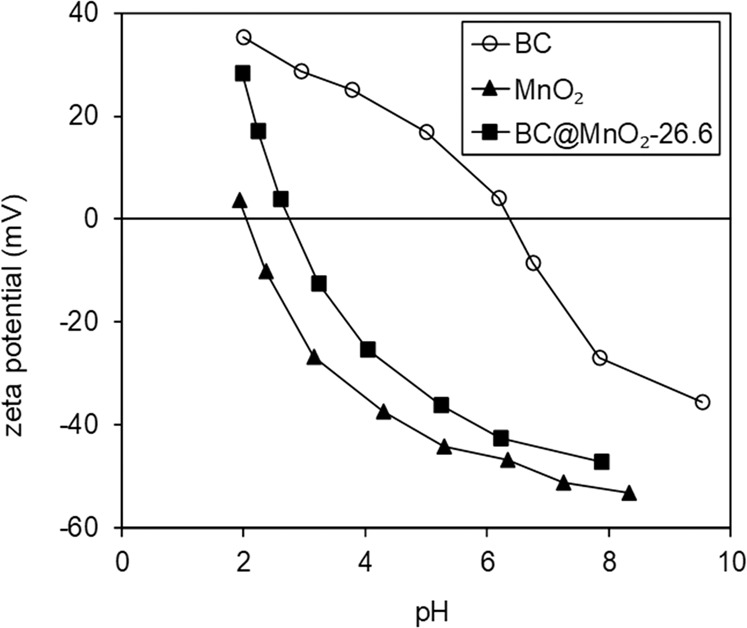
Zeta potentials of BC, MnO_2_ and BC@MnO_2_-26.6 as a function of solution pH.

The surface zeta potentials of BC, MnO_2_, and BC@MnO_2_-26.6 as a function of solution pH are shown in Fig. 4. The zeta potentials of the adsorbents monotonically decreased with increasing pH due to continuous de-protonation of surface hydroxide groups. The isoelectric point (IEP) of BC was 6.4. The deposition of MnO_2_ caused a significant decrease in the zeta potential, and the IEP of BC@MnO_2_-26.6 was 2.8. Notably, the IEP of MnO_2_ was 2.1, consistent with a prior report (2–4)^8^. Hence, the lower IEP of BC@MnO_2_-26.6 compared to that of MnO_2_ may be due to the deposition of MnO_2_.

### Effect of solution pH

**Figure 5:**
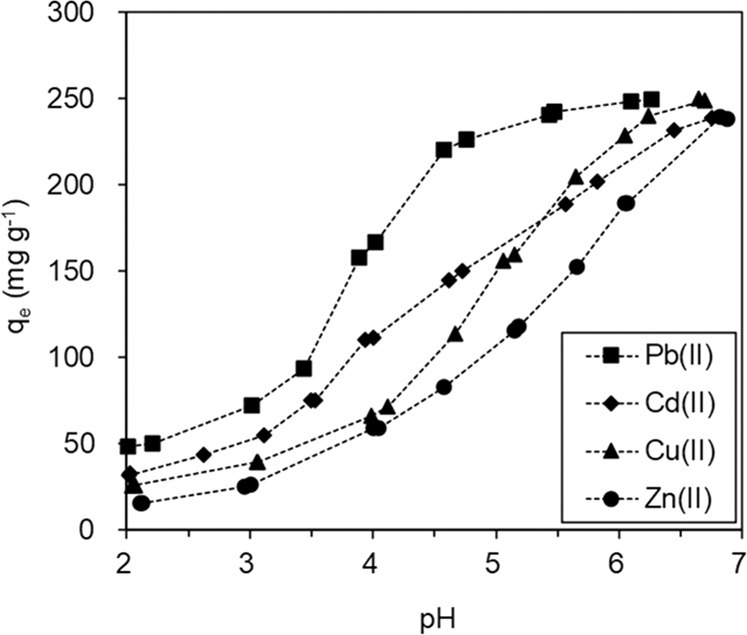
Influence of solution pH on Pb(II), Cd(II), Cu(II) and Zn(II) adsorption to BC@MnO_2_-26.6.

The solution pH can affect the formation of heavy metal ions and the surface charges of adsorbents in aqueous solution, and so influences the adsorption of heavy metal ions. We explored the impact of solution pH (2–7) on the adsorption of heavy metal ions on BC@MnO_2_-26.6 (Fig. 5). The solution pH exerted a marked impact on the adsorption of heavy metal ions on BC@MnO_2_-26.6. The Cd(II), Cu(II), Zn(II), and Pb(II) adsorption capacity gradually decreased with decreasing solution pH. A reduced adsorption capacity for heavy metal ions at lower pH values has been reported by others^9,10,14^. This may be because the surface functional groups of BC@MnO_2_-26.6 are protonized at low pH, producing electrostatic repulsion between free heavy metal ions and the positively charged surface functional groups. Also, a low solution pH triggers competition for adsorption sites between heavy metal ions and hydrogen ions, leading to low adsorption capacities of 33.5, 26.0, 15.9, and 48.2 for Cd(II), Cu(II), Zn(II), and Pb(II) respectively, at pH ~2.0.

By contrast, the competition between heavy metal ions and hydrogen ions is reduced at higher pH, facilitating the adsorption of heavy metal ions. The IEP of BC@MnO_2_-26.6 was 2.8, and the surface functional groups of BC@MnO_2_-26.6 were deprotonated at pH > 2.8. This led to a more negative surface charge at solution pH > 2.8, thereby enhancing the electrostatic attraction between the surface of BC@MnO_2_-26.6 and heavy metals, and thus improving adsorption capacity. The peak adsorption capacities of BC@MnO_2_-26.6 were 232.5, 248.9, 239.4, and 249.2 mg g^−1^ for Cd(II), Cu(II), Zn(II), and Pb(II), respectively, at pH > 6.5. Although precipitation influenced the removal of heavy metal ions from aqueous solution by adsorbents, pH 4.5 was optimal and was used in subsequent experiments.

### Absorption kinetics

**Figure 6:**
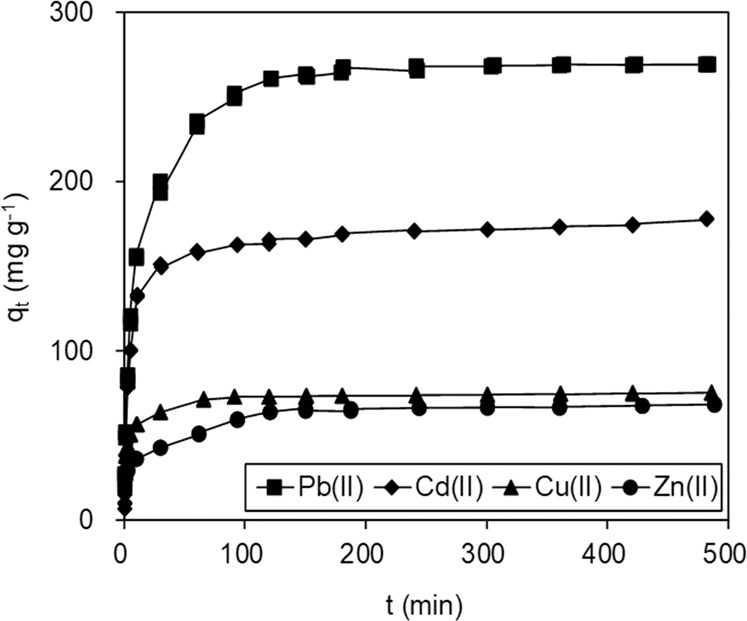
Kinetics of Pb(II), Cd(II), Cu(II) and Zn(II) adsorption onto BC@MnO_2_-26.6.

The kinetics of adsorption of heavy metal ions by BC@MnO_2_-26.6 were investigated (Fig. 6) to identify the contact time that resulted in equilibrium adsorption. Cd(II), Cu(II), Zn(II), and Pb(II) adsorption on BC@MnO_2_-26.6 increased rapidly in the first 60 min and subsequently declined gradually to reach an equilibrium at 120 min. To further evaluate the adsorption of heavy metal ions, pseudo-first-order^29^ and pseudo-second-order^30^ models were applied. The linear kinetic equations are presented in the Supporting Information.

The fitted curves obtained using pseudo-first-order and pseudo-second-order models are presented in Fig. S2a,b, and the resulting calculation parameters are listed in Table 2. The low *R*2 values indicate that the pseudo-first-order model did not reflect the adsorption of the four heavy metal ions onto BC@MnO_2_-26.6. When using the pseudo-first-order model, it was assumed that the adsorption capacity is proportional to the difference between the capacity at any time *t* (*q*_*t*_) and the maximum capacity at equilibrium (*q*_*e*_)^31^, which is typically satisfactory at the beginning of the reaction but not during the entire contact time. The pseudo-second-order model was more suitable, as indicated by the high *R*2 values and the consistent *q*_*exp*_ and *q’*_*cal*_ values. That model assumes that the rate-limiting step is chemisorption or chemical absorption, which involves valency forces generated by the exchange or sharing of electrons between the adsorbate and adsorbent^32,33^.

**Table 2:** Fitting parameters of Pb(II), Cd(II), Cu(II) and Zn(II) adsorption kinetics using pseudo-first-order and pseudo-second-order model.

Sorbents	*q*_exp_ mg g^−1^	Pseudo-first-order model			Pseudo-second-order model		
		*k*_1_ min^−1^	*q*_cal_ mg g^−1^	*R*2	*k*_2_ g mg^−1^ min^−1^	*q*′_cal_ mg g^−1^	*R*2
Pb(II)	268.9	0.0159	122.1	0.958	0.0005	270.3	0.999
Cd(II)	178.2	0.0085	69.1	0.825	0.0008	178.6	0.999
Cu(II)	75.2	0.0090	18.2	0.798	0.0046	75.2	0.999
Zn(II)	68.6	0.0092	31.3	0.905	0.0016	69.0	0.999

To gain further insight into the adsorption of Pb(II), Cd(II), Cu(II), and Zn(II) onto BC@MnO_2_-26.6, the Weber-Morris model was adopted. The results fitted using the Weber-Morris model are shown in Fig. S2c, and the calculated parameters are listed in Table 3. The curves for Pb(II), Cd(II), Cu(II), or Zn(II) adsorption contained three linear portions, suggesting that their adsorption involved multiple steps^34^. The first linear portion can be attributed to diffusion of heavy metal ions to the external surface of BC@MnO_2_-26.6 and the large-pore region of BC, the second to diffusion of heavy metal ions into the small pores of BC and nano-MnO_2_ aggregates, and the third to the final equilibrium stage. A similar diffusion pattern was observed for heavy-metal uptake by Al_2_O_3_-pillared manganese oxide and porous material-supported MnO_2_ by us and another group^9,31^.

**Table 3:** Fitting parameters of heavy metal ions adsorption kinetics using Weber-Morris model.

Ion	*C*_0_ mg L^−1^	Weber-Morris model					
		*k*_1_ mg g^−1^ min^−1/2^	*I*_1_	*R*2	*k*_2_ mg g^−1^ min^−1/2^	*I*_2_	*R*2
Pb(II)	250	48.7	3.5	0.993	11.6	137.2	0.956
Cd(II)	100	32.3	5.4	0.965	40.7	1.2	0.987
Cu(II)	250	32.5	6.4	0.997	4.8	38.5	0.925
Zn(II)	250	19.1	6.2	0.956	3.6	23.5	0.993

### Adsorption isotherms

**Figure 7:**
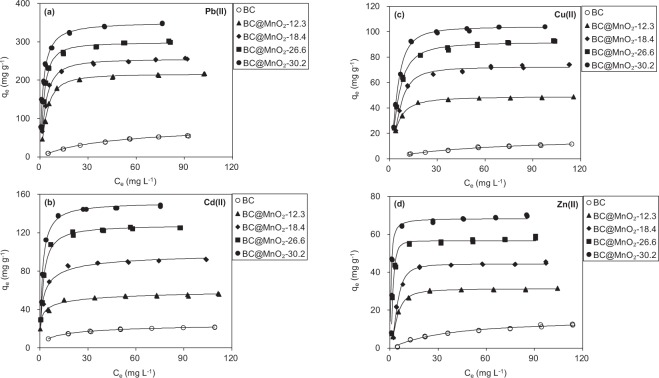
Isotherms of Pb(II), Cd(II), Cu(II) and Zn(II) adsorption onto adsorbents.

The isotherms representing the adsorption of Pb(II), Cd(II), Cu(II), and Zn(II) onto the adsorbents from aqueous solution are shown in Fig. 7a–d, respectively. The adsorption capacity of BC for Pb(II), Cd(II), Cu(II), and Zn(II) was very low. Consistently, biochar adsorbents exhibited low capacities for adsorbing heavy metal ions in prior works^31^. By contrast, MnO_2_ loading significantly enhanced the adsorption capacities for Cd(II), Cu(II), Zn(II), and Pb(II), reflecting the crucial role of MnO_2_ in the adsorption of heavy metal ions. Notably, BC@MnO_2_-30.2 exhibited a greater capacity for adsorbing heavy metal ions than BC@MnO_2_-12.3, BC@MnO_2_-18.4, or BC@MnO_2_-26.6, likely due to its high MnO_2_ content. To explore the mechanism of adsorption, the Langmuir isotherm model was adopted to fit the adsorption isotherms of adsorbents (Table 4). The Langmuir adsorption model (*R*^2^ > 0.9) explained Pb(II), Cd(II), Cu(II), and Zn(II) adsorption well, indicating that the adsorption sites were evenly distributed^35^. The calculated maximum adsorption capacities (*q*_*m*_) of BC@MnO_2_ for Pb(II), Cd(II), Cu(II), and Zn(II) were 216.22–351.37, 66.48–151.43, 48.90–103.91, and 31.25–68.36 mg g^−1^, respectively, markedly higher than those of BC. These results confirmed that the heavy metal ions adsorption capacities of BC were greatly increased by MnO_2_ deposition. In addition, the MnO_2_-loaded BC adsorbents had large *b* values in the Langmuir model, suggesting high adsorption affinity and selectivity for heavy metal ions.

**Table 4:** Fitting parameters of Pb(II), Cd(II), Cu(II) and Zn(II) adsorption isotherms on the adsorbents using Langmuir isotherm model.

Sorbents		Langmuir isotherm model			
		*b* (L g^−1^)	*q*_m_ (mg g^−1^)	*Q*_m_ (mg gMnO_2_^−1^)	*R*2
BC	Pb(II)	0.02	82.50	—	0.998
	Cd(II)	0.18	26.51	—	0.994
	Cu(II)	0.02	19.14	—	0.985
	Zn(II)	0.02	15.14	—	0.976
BC@MnO_2_-12.3	Pb(II)	0.12	216.22	1757.89	0.996
	Cd(II)	0.86	66.48	540.49	0.984
	Cu(II)	0.11	48.90	397.56	0.995
	Zn(II)	0.07	31.25	254.07	0.998
BC@MnO_2_-18.4	Pb(II)	0.19	255.45	1388.32	0.994
	Cd(II)	0.64	100.76	547.61	0.976
	Cu(II)	0.03	72.28	392.83	0.991
	Zn(II)	0.03	44.40	392.83	0.993
BC@MnO_2_-26.3	Pb(II)	0.27	298.27	1134.11	0.992
	Cd(II)	0.50	127.95	486.50	0.976
	Cu(II)	0.06	91.67	348.56	0.996
	Zn(II)	0.22	56.60	215.21	0.963
BC@MnO_2_-30.2	Pb(II)	0.49	351.37	1163.48	0.986
	Cd(II)	0.62	151.43	501.42	0.983
	Cu(II)	0.06	103.91	344.07	0.998
	Zn(II)	0.23	68.36	226.36	0.983

**Figure 8:**
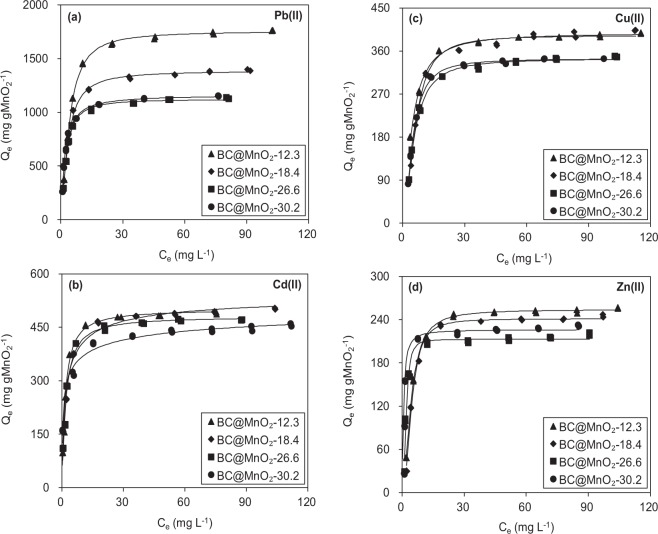
MnO_2_ mass normalized Pb(II), Cd(II), Cu(II) and Zn(II) adsorption isotherms on the samples.

The mass of MnO_2_ was used to normalize the adsorption isotherms. The results are shown in Fig. 8a–d, and the fitting parameters are listed in Table 4. For Pb(II) (Fig. 8a), the normalized adsorption capacities (*Q*_*m*_) were 1757.89, 1388.32, 1134.11, and 1163.48 mg g·MnO_2_^−1^ for BC@MnO_2_-12.3, BC@MnO_2_-18.4, BC@MnO_2_-26.6, and BC@MnO_2_-30.2, respectively. Therefore, the normalized Pb(II) adsorption capacity (*Q*_*m*_) of the BC@MnO_2_ adsorbents decreased with increasing MnO_2_ load. Consistently, adsorption onto BC@MnO_2_ decreased in the following order: Cd(II) (Fig. 8b), Cu(II) (Fig. 8c), and Zn(II) (Fig. 8d). These results indicated that the utilization efficiency per unit mass MnO_2_ of BC@MnO_2_ decreased with increasing MnO_2_ deposition. As reported recently by us and others^36,37^, the lower the load of active moieties, the better the dispersibility on the surface of the support. Moreover, excessive Mn leads to generation of multi-layer MnO_2_ moieties (as shown by TEM Fig. 2), which reduces the accessibility of the inner layer of MnO_2_. BC@MnO_2_-12.3 had a higher MnO_2_-normalized heavy metal ion adsorption capacity than the other BC@MnO_2_ adsorbents.

### Effect of coexisting ions

**Figure 9:**
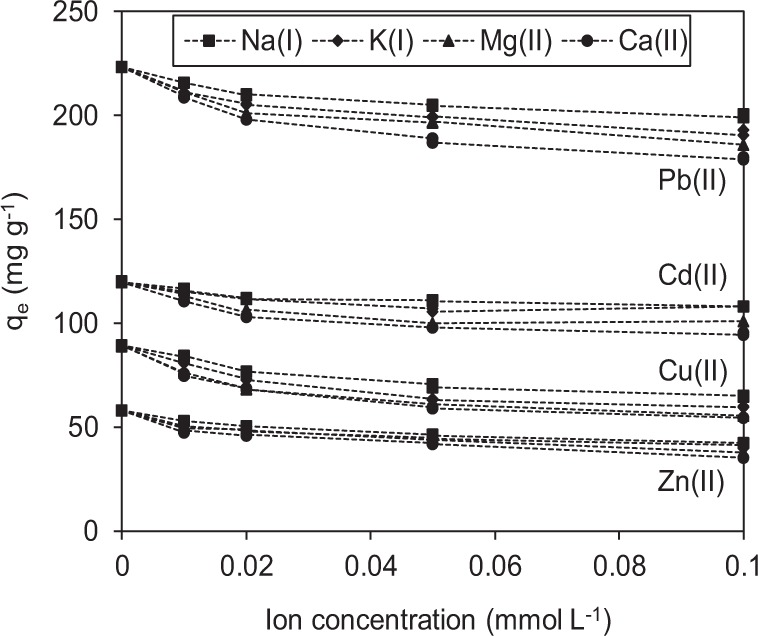
Influence of co-existing ions on Pb(II), Cd(II), Cu(II) and Zn(II) adsorption to BC@MnO_2_-26.6.

K(I), Na(I), Ca(II), and Mg(II) are natural cations and common alkaline-earth metal cations in wastewater or natural water. K(I), Na(I), Ca(II), and Mg(II)) were used as coexisting ions to assess the absorption selectivity of BC@MnO_2_-26.6 (Fig. 9) and BC (Fig. S3) for Cd(II), Cu(II), Zn(II), and Pb(II). As the coexisting ion concentration increased, the adsorption capacity of BC@MnO_2_-26.6 and BC for Cd(II), Cu(II), Zn(II), and Pb(II) decreased. Therefore, an increased concentration of coexisting ions at the solid/liquid interface has a considerable impact on the adsorbate–adsorbent interaction. This may be caused by a screening effect of electrostatic interactions between the adsorbate and adsorbent, or by competition between the coexisting cations and heavy metal ions for negatively charged adsorption sites. In addition, an increased concentration of coexisting ions reduces the interface potential and thickness of the electric double layer, reducing electrostatic adsorption^38^. However, compared to BC, BC@MnO_2_-26.6 exhibited greater adsorption of Pb(II), Cd(II), Cu(II), and Zn(II) as the coexisting cation concentration increased from 0.01 to 0.1 mmol L^−1^. The adsorption capacity of BC for Pb(II), Cd(II), Cu(II), and Zn(II) decreased by 0.9–77.7%, 22.0–71.4%, 11.6–60.7% and 9.7–63.9%, respectively, compared to 3.4–19.8%, 3.2–21.4%, 5.2–38.8%, and 8.6–38.7%, respectively, for BC@MnO_2_-26.6. The higher adsorption capacity of BC@MnO_2_-26.6 was due to the inner sphere between MnO_2_ and the target metal ions, which is more selective than the nonspecific outer sphere comprising oxygen-containing BC groups. Inner-sphere complexation of heavy metal ions with manganese oxides has been confirmed using extended X-ray absorption fine structure (XAFS) and XAFS spectroscopy^39,40^. We next explored the mechanism of absorption using XPS.

### Adsorption mechanism

**Figure 10:**
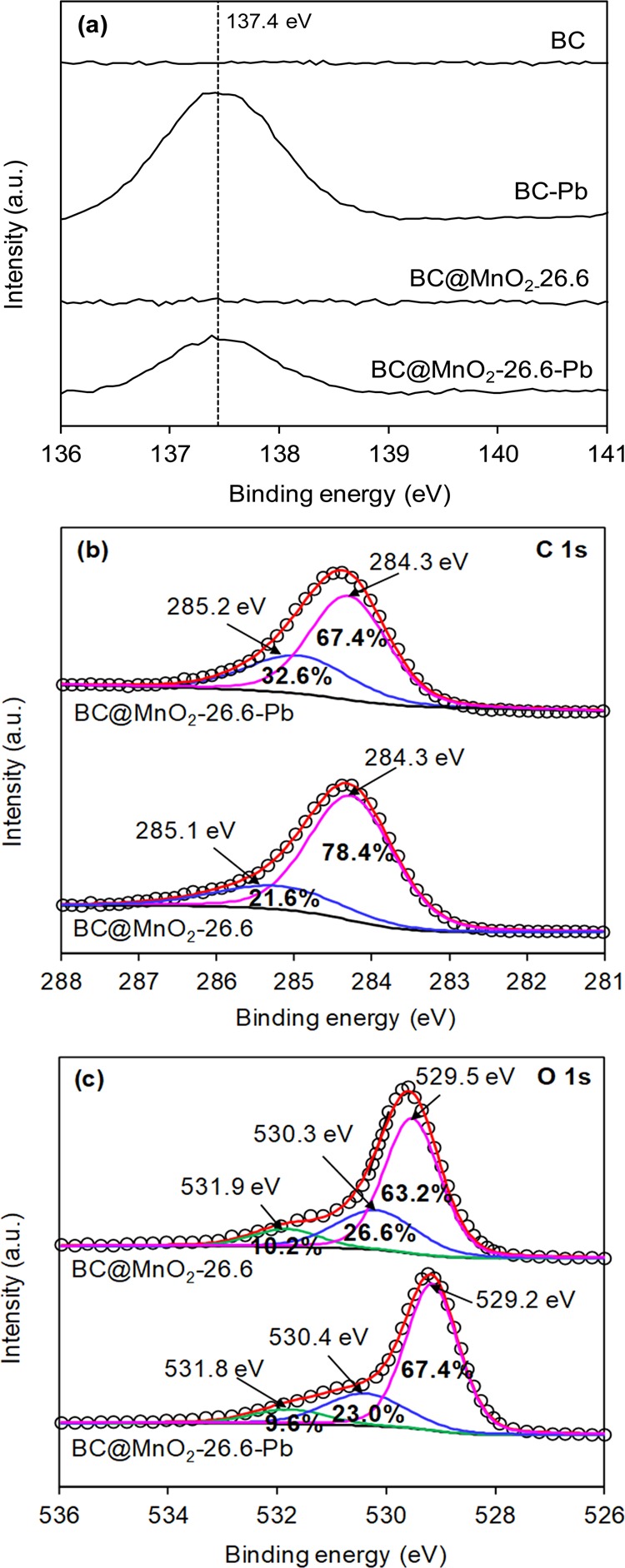
High-resolution XPS spectra of (**a**) Pb 4f_7/2_, (**b**) C 1 s and (**c**) O 1 s of BC@MnO_2_-26.6 before and after Pb(II) adsorption.

To determine the mechanism of heavy metal ions adsorption onto BC@MnO_2_, the XPS spectra of BC and BC@MnO_2_-26.6 before and after adsorption of Pb(II) were analyzed (Fig. 2). Pb 4 f peaks were detected for BC and BC@MnO_2_-26.6 after Pb(II) adsorption. The Pb 4f_7/2_ XPS spectra of BC and BC@MnO_2_-26.6 before and after Pb(II) adsorption are shown in Fig. 10a. After Pb(II) adsorption, BC and BC@MnO_2_-26.6 produced Pb 4f_7/2_ peaks at a binding energy of 137.4 eV, corresponding to an orthorhombic PbO compound^41^. No carbonate or hydroxide of Pb was formed during adsorption. This indicates that Pb(II) was adsorbed onto BC and BC@MnO_2_-26.6 via carbonyl and hydroxyl groups.

The detailed XPS spectra of Cu 2p, Cd 3d and Zn 2p of BC@MnO_2_-26.6 after Cu(II), Cd(II) and Zn(II) adsorption were also analyzed, and the results are shown in Fig. S4. After Cu(II) adsorption, the binding energy of 932.4 eV for Cu 2p in the spectrum of BC@MnO_2_-26.6 can be attributed to Cu 2p_3/2_, indicating the existence of cuprous and cupric forms in BC@MnO_2_-26.6^42^. As shown in Fig. S4a, the weak satellite peaks observed in the spectrum were assigned as Cu 3p electron, suggesting the presence of CuMn_2_O_4_ on BC@MnO_2_-26.6 after adsorption^42^. The absorption peaks of Cd 3d (Fig. S4b) and Zn 2p (Fig. S4c) appear in the XPS spectra of BC@MnO_2_-26.6 after Cd(II) and Zn(II) adsorption, respectively, demonstrating the successful adsorption of Cd(II) and Zn(II) by the adsorbent. The peaks of Cd 3d and Zn 2p can be ascribed to CdO and ZnO, respectively, because of the consistency between the measured value and reported value^43,44^. This explains the mechanism of Cd(II) and Zn(II) removal by BC@MnO_2_-26.6 adsorbent.

The detailed C1s XPS spectra of BC@MnO_2_-26.6 showed two main peaks at 284.3 and 285.2 eV (Fig. 10b), which corresponded to C-C and C-O groups^45^, respectively. After Pb(II) adsorption, the binding energies of the C-C and C-O groups were not changed significantly, suggesting that the existing forms of C in BC@MnO_2_-26.6 were not affected by Pb(II) binding. However, the C-O ratio in BC@MnO_2_-26.6 increased after Pb(II) adsorption, possibly due to the formation of C-O-Pb at the adsorption sites^46^.

To validate the mechanism of heavy metal ions adsorption onto BC@MnO_2_, the O1s XPS spectra of BC@MnO_2_-26.6 before and after adsorption of Pb(II) were evaluated (Fig. 10c). Before and after Pb(II) adsorption, the high-resolution O1s XPS spectra of BC@MnO_2_-26.6 showed an obvious tail and a wide shoulder at high binding energy. Based on previous reports, a O1s spectrum consists of three peaks that correspond to different forms of oxygen^47^: hydroxide oxygen (OH^−^), lattice oxygen (O^2−^), and oxygen and hydroxides in molecular water (*i.e*., chemisorbed, physisorbed, and structural H_2_O, and water not securely attached to the surface in terms of electrical contact). We used matched these forms to peaks in the O1s spectra (Table 5). After Pb(II) adsorption, the binding energies of hydroxide oxygen (OH^−^), lattice oxygen (O^2−^), and oxygen in molecular water did not change significantly, suggesting that the existing forms of O in BC@MnO_2_-26.6 were not affected by Pb(II) binding. However, after Pb(II) binding, the peak area for hydroxide oxygen (OH^−^) in BC@MnO_2_-26.6 decreased from 26.6% to 23.0%. By contrast, the peak at 529.2–529.5 eV, assigned to M-O-Pb (M=C or Mn), increased from 63.2% to 67.4% after Pb(II) adsorption, due to the generation of more bidentate binuclear, bidentate mononuclear, and multidentate complexes than monodentate complexes during the adsorption of Pb(II). According to the model of adsorption, multidentate or bidentate complexes embed two or three OH^−^ groups on the adsorbent surface, unlike monodentate complexes^17^. In addition, OH^−^ groups on the surface connect to metal centers and generate dentate complexes.

**Table 5:** The results of XPS C 1 s and O 1 s multiplets peak fitting.

Sorbents	C-C			C-O			O^2−^			OH^−^			H_2_O		
	BE^a^ (eV)	FWHM^b^ (eV)	At.^c^ (%)	BE (eV)	FWHM (eV)	At. (%)	BE (eV)	FWHM (eV)	At. (%)	BE (eV)	FWHM (eV)	At. (%)	BE (eV)	FWHM (eV)	At. (%)
BC@MnO_2_-26.6	284.3	1.28	78.4	285.2	1.88	21.6	529.5	1.20	63.2	530.2	1.73	26.6	531.8	1.47	10.2
BC@MnO_2_-26.6-Pb	284.3	1.18	67.4	285.1	1.56	32.6	529.2	1.14	67.4	530.4	1.76	23.0	531.8	1.63	9.6

^a^Binding energy.

^b^The FWHM of all peaks were constrained.

^c^At. represents the percentage of the contribution for each peak to the total number of counts under the Mn 2p_3/2_ or O 1 s peak, and all peaks modeled as 70% Gaussian-30% Lorentzian.

BC supports MnO_2_ and facilitates its non-specific complexation with heavy metal ions. The negatively charged, non-diffusible, oxygen-containing groups (*e.g*., CO^−^ and COO^−^) that are covalently bound to BC (exchange capacity 76.3 mmol kg^−1^ at pH 6) may be the main actors within the BC@MnO_2_ in the enhancement of pre-concentration and permeation of target metal cations from solution, instead of metal cations being captured by the loaded MnO_2_. In addition, the heavy metal ions also could be adsorbed by the functional groups (*e.g*., -COOH and -COH) on BC surface^17^. The adsorption took place due to the elimination of H^+^ through -COOH and -COH functional groups into aqueous solution, forming C=O-O-Pb and C-O-Pb. This is consisted with the C 1 s XPS results.

### Fixed-bed column sorption

**Figure 11:**
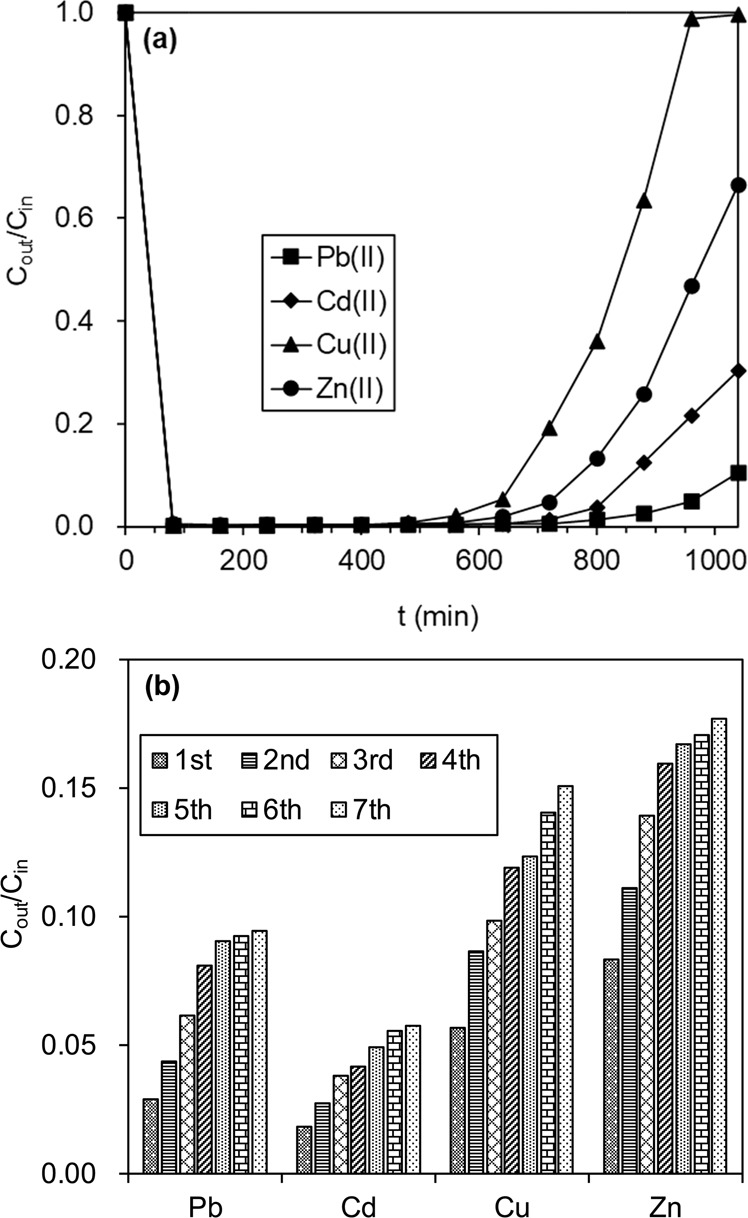
(**a**) Removal capacity of heavy metals by BC@MnO_2_-26.6 column and (**b**) Multiple cycles of adsorption-desorption through BC@MnO_2_-26.6 column.

A fixed-bed column sorption test of BC@MnO_2_-26.6 was conducted to investigate the potential of BC@MnO_2_ for applications in engineering; the breakthrough curves are shown in Fig. 11a. At an adsorption time of 600 min, the removal rates of Pb(II), Cd(II), Cu(II), and Zn(II) were >98%. The removal rates decreased with increasing adsorption time. Similar results have been reported for other MnO_2_ systems^8,31^. The treatable bed volume (BV) of Pb(II), Cd(II), Cu(II), and Zn(II) by BC@MnO_2_-26.6 was 320, 233, 267, and 213 BV, respectively. Because we did not investigate the column adsorption capacity, the Thomas model was adopted to predict column breakthrough (at *C*_*e*_/*C*_0_ = 1)^48^. The removal capacity at the breakthrough point was 2.1 × 10^6^, 1.1 × 10^6^, 6.7 × 10^5^, and 5.2 × 10^5^ mg g^−1^ for Pb(II), Cd(II), Cu(II), and Zn(II), respectively, significantly higher than for commercial BCs, nano-MnO_2_, or MnO_2_-loaded resin^8,46,49^. This is mainly due to the highly dispersed MnO_2_ on the surface of the BC. Therefore, BC@MnO_2_-26.6 is highly effective for removing heavy metal ions from water in fixed-bed and batch modes and is suitable for *in situ* environmental remediation.

The exhausted BC@MnO_2_-26.6 was regenerated *in situ* using 150 mL of 0.1 mol L^−1^ HNO_3_ and rinsed with 120 mL of distilled water at 298 K. After regeneration, the concentration of Pb(II), Cd(II), Cu(II), and Zn(II) in effluent was <0.001 mg L^−1^. To investigate the reusability of BC@MnO_2_-26.6, the fixed-bed column was subjected to seven adsorption-desorption cycles (Fig. 11b). In the first adsorption-desorption cycle, the removal efficiency of the fixed-bed column for Pb(II), Cd(II), Cu(II), and Zn(II) decreased by 6.4%, 4.4%, 8.7%, and 3.1%, respectively, while the decrease was less than 1.0% after five cycles. Therefore, the performance of the BC@MnO_2_-26.6 fixed-bed column for adsorption of heavy metal ions was highly stable.

A sample of wastewater from a Chinese electroplating plant in Yangzhou City was used as the influent to verify the feasibility of BC@MnO_2_-26.6 for decontaminating industrial wastewater. After treatment in a BC@MnO_2_-26.6 column for 10 h, the Pb(II), Cd(II), Cu(II), and Zn(II) concentrations in effluent were 0.002, 0.009, 0.007, and 0.004 mg L^−1^, respectively. The removal rates of total Pb(II), Cd(II), Cu(II) and Zn(II) were >99.1%. Additionally, the pH and COD of the effluent were 5.23 and 23.6 mg L^−1^, respectively. The pH of wastewater increased slightly, as reported for MnO_2_ adsorbents for purifying electroplating wastewater^45,50^. The COD in effluent decreased by 97.3% compared to that in influent, suggesting removal of COD by the BC@MnO_2_-26.6 column. Therefore, the BC@MnO_2_-26.6 column shows promise for remediation of electroplating wastewater in terms of removing heavy metal ions contaminants.

## Conclusion

This study provides an effective approach to enhance heavy metal ions adsorption by biochar derived from water hyacinth through loading MnO_2_ on the its surface. In comparison to the blank biochar, high adsorption capacities for heavy metal ions were observed for MnO_2_-loaded biochar as a result of its abundant surface Mn-OH groups. Increasing MnO_2_ loading amount led to enhanced heavy metal ions adsorption, whereas the MnO_2_-normalized adsorption amounts remained nearly identical at MnO_2_ loads of 26.6% to 30.2%. XPS analyses revealed that the surface complexation is proposed as the dominant mechanism responsible for heavy metal ions immobilization by MnO_2_-loaded biochar. The column sorption and regeneration tests using simulated and real wastewater indicated that MnO_2_-loaded biochar could be used as a highly effective adsorbent for heavy metal ions removal in water.

## Methods

### Materials and reagents

Chemicals of analytical grade or higher were used directly without further purification (Aladdin Industrial Corporation). Distilled deionized water (resistivity >18.2 MΩ cm^−1^) was used to prepare all solutions. Nitrate salts of heavy metal ions were dissolved and diluted to prepare heavy metal ion stock solutions (1 g L^−1^). Water hyacinth, one of the most aggressive invasive species of aquatic plants worldwide^51^, was selected as the feedstock for BC and collected from Yiyang River, Yangzhou, China.

### Preparation of adsorbents

Water hyacinth plants were washed repeatedly in deionized water to remove impurities and dried at 80 °C for 24 h. The dried water hyacinth was milled and passed through a 0.2 mm sieve. Slow pyrolysis was performed to prepare water hyacinth-based BC at a low temperature under oxygen-limited conditions. In short, 2 g of water hyacinth were placed in a porcelain crucible and transferred to a muffle furnace, the temperature of which was increased from room temperature to 450 °C at 5°C min^−1^ and maintained for 3 h. After heating, the muffle furnace was cooled to room temperature and the resulting solid was passed through a 0.154 mm sieve.

Nanosized MnO_2_-loaded BCs were prepared using potassium permanganate (KMnO_4_) as the source of manganese. Briefly, 4 g of BC were placed in 100 mL of deionized water at 25 °C, and the desired volume of KMnO_4_ solution was added with stirring at 25 °C for 30 min. MnO_2_-loaded BC was synthesized via the dropwise addition of 40 mL of 30% H_2_O_2_ with vigorous stirring. Next, the pH was adjusted to 7.0 using 1.0 mol L^−1^ HNO_3_. The solution was stirred for 30 min and stored at room temperature for 3 h. The material was recovered by filtration, rinsed several times using deionized water, and dried at 105 °C for 12 h. In this way, BC@MnO_2_-X was prepared, where X refers to the MnO_2_ deposition amount (% wt.).

### Adsorbent characterization

A JEM-2100 transmission electron microscope (JEOL, Japan) was used to visualize the morphology of the adsorbents. A Rigaku *D*/max-RA powder diffractometer (Rigaku, Japan) equipped with a Cu Kα radiation source was used to determine the XRD patterns from various angles (20–80°). An ARL9800XP X-ray fluorescence spectrometer (Thermo Electron Corp., Switzerland) was used to determine the MnO_2_ content in the adsorbents. The testing adsorbent was pressed into thinner disc and determined directly using semi quantitative method. The specific surface areas and pore volumes of the samples were determined using a Micromeritics ASAP 2020 analyzer (Micromeritics Instrument Co., Norcross, GA, USA) via N_2_ adsorption-desorption measurements at −196 °C (77 K). Using monochromatized Al Kα (where *hv* is 1486.6 eV) as a source, the samples were subjected to XPS. The C1s peak at 284.6 eV was used to calibrate the binding-energy values. A zeta potential analyzer (Brookhaven Instruments Ltd., USA) was used to measure the surface zeta potentials. The Mn(IV) content in BC@MnO_2_ adsorbent was measured by using oxalic acid-permanganate back-titration method^47^, and the determination procedures are detailed in Supporting Information.

### Batch adsorption experiments

The adsorption isotherms of Cd(II), Cu(II), Zn(II), and Pb(II) by BC and BC@MnO_2_ at 25 °C were investigated in batch experiments. Briefly, 20 mg of blank biochar or MnO_2_-loaded biochar was added into 40-ml of glass vials with polytetrafluoroethylene-lined screw caps receiving 40 ml Pb(NO_3_)_2_, Cd(NO_3_)_2_, Cu(NO_3_)_2_ or Zn(NO_3_)_2_ solution with predetermined concentrations. The initial concentration of heavy metal ions was 5–200 mg/L. HNO_3_ or NaOH (0.1 mol L^−1^) was used to adjust the pH to 4.5. The suspensions were then mixed end over end using the rotary shaker for 24 h. According to the preliminarily kinetic tests, an adsorption equilibrium was achieved after shaking for 24 h. The equilibrium solutions were passed through a 0.22-μm membrane, and atomic absorption spectrometry (AAS) (Perkin Elmer 2380, USA) was performed to calculate the residual solute concentration. The adsorption of heavy metal ions was calculated on a mass-balance basis.

To assess the adsorption kinetics of heavy metal ions, 250 mg of adsorbent was added to 500-mL flasks containing 500 mL of 150 mg L^−1^ Cd(NO_3_)_2_, 120 mg L^−1^ Pb(NO_3_)_2_, 120 mg L^−1^ Cu(NO_3_)_2_ or 80 mg L^−1^ Zn(NO_3_)_2_ solution with magnetic stirring at pH 4.5 and 25 °C. At each time point, 5 mL of sample were removed from each flask.

To determine the effect of pH on adsorption, 25 mg of adsorbent were dispersed in 40 mL of 150 mg L^−1^ Pb(NO_3_)_2_, Cd(NO_3_)_2_, Cu(NO_3_)_2_ or Zn(NO_3_)_2_ solution at pH 2–7 and 25 °C. The pH was adjusted by adding negligible volumes of 0.1 mol L^−1^ HNO_3_ or NaOH. To determine the effect of coexisting ions on adsorption, 20 mg of BC@MnO_2_-26.6 or BC were dispersed in 40 mL of NaNO_3_, KNO_3_, Mg(NO_3_)_2_, or Ca(NO_3_)_2_ solution (0.01–0.1 mmol/L) containing 120 mg/L Pb(NO_3_)_2_, 150 mg/L Cd(NO_3_)_2_, 120 mg/L Cu(NO_3_)_2_, or 80 mg/L Zn(NO_3_)_2_ at pH 4.5 and 25 °C. The adsorption data were collected in duplicate, and mean values were calculated.

### Column adsorption and regeneration tests

Column adsorption tests of the removal of heavy metal ions were carried out at 25 °C using a polyethylene column (Omnifit Co., UK) with an inner diameter of 15 mm and a length of 130 mm containing BC@MnO_2_-26.6. Three milliliters of wet BC@MnO_2_-26.6 powder (4.5 g) were packed in the column. At the top and bottom of the column, quartz sand (diameter ~0.2 mm; does not adsorb heavy metal ions) was packed to prevent the loss of adsorbents and control the flow. Four synthetic wastewaters containing 75 mg L^−1^ Pb(II), 60 mg L^−1^ Cd(II), 40 mg L^−1^ Cu(II) or 40 mg L^−1^ Zn(II), respectively, were used as influents in the column adsorption tests. Pb(II), Cd(II), Cu(II) and Zn(II) were removed from synthetic wastewater by four separate column beds packed with the same mass of BC@MnO_2_-26.6 powder. The breakthrough points were set as 0.1 mg L^−1^ for Pb(II), 0.01 mg L^−1^ for Cd(II), 1.5 mg L^−1^ for Cu(II), and 5.0 mg L^−1^ for Zn(II), as the permitted maximum concentrations by China’s Quality Standard for Ground Water (GB/T 14848-2017). The influents continuously pumped through the column at a rate of 60.0 mL h^−1^ using a calibrated syringe pump (Longerpump Co., China) for 10 h. Effluent samples were collected regularly using a BSZ-100 fraction collector (Huxi Analysis Instrument Factory Co., China) and subjected to AAS. Next, 0.1 mol L^−1^ HNO_3_ solution was added (30.0 mL h^−1^ for 5 h in the upwards direction) to exhaust the column, followed by rinsing with 120 mL of distilled water. The procedure for the first adsorption-desorption cycle was used for the following seven cycles, and the total adsorption time was 10 h.

To investigate the feasibility of BC@MnO_2_-26.6 for removing heavy metal ions from wastewater, we collected sewage from the sewage discharge port of an electroplating factory in Yangzhou. The wastewater was used directly without treatment. The pH and chemical oxygen demand (COD) were 4.51 and 864.7 mg L^−1^, respectively. The concentrations of Pb(II), Cd(II), Cu(II), and Zn(II) in the wastewater were 25.6, 57.1, 11.3, and 14.7 mg L^−1^, respectively.

## Supplementary information

Supplementary information.
