# Supplementary material for: Enhanced removal of heavy metal ions from aqueous solution using manganese dioxide-loaded biochar: Behavior and mechanism
Source: Sci Rep. 2020 Apr 8;10:6067. doi: 10.1038/s41598-020-63000-z (PMC7142110; doi:10.1038/s41598-020-63000-z)
Supplement: Supplementary file 1 — Supplementary information. [file 41598_2020_63000_MOESM1_ESM.pdf]

Supporting Information (SI)

Enhanced removal of heavy metal ions from aqueous solution using manganese dioxide-loaded biochar: Behavior and mechanism

Haipeng Zhang[a,b], Fangfang Xu[b], Jinyuan Xue[b], Shiyong Chen[b], Juanjuan Wang[b], Yanju Yang[b,*]

[a] *Jiangsu Key Laboratory of Crop Genetics and Physiology, Jiangsu Co-Innovation Center for Modern Production Technology of Grain Crops, Yangzhou University, Yangzhou 225009, China*

[b] *College of Environmental Science and Engineering, Yangzhou University, Yangzhou 225009, China*

The SI contains 12 total pages with 4 Figures.

*Corresponding author:
E-mail address: yangyanju@yzu.edu.cn (Y.J. Yang).

Figure captions:

Figure S1: (a) N_2 adsorption-desorption isotherms and (b) pore size distributions of the samples.

Figure S2: Fitting kinetics of heavy metal ions adsorption onto BC@MnO_2-26.6 using (a) the pseudo-first-order model, (b) the pseudo-second-order model and (c) the Webber-Morris model.

Figure S3: Effect of co-existing ions on heavy metal ions onto BC.

Figure S4: XPS spectra of BC@MnO_2-26.6 after the sorption of Cu(II), Cd(II) and Zn(II). (a) Cu 2p, (b) Cd 3d and (c) Zn 2p.

Sorption kinetic models:

The kinetic data of Pb(II), Cd(II), Cu(II) and Zn(II) sorption onto BC@MnO_2-26.6 were analyzed using two widely used kinetic models, the pseudo-first-order model, the pseudo-second-order model and the Webber-Morris model. The pseudo-first-order kinetic model is described as equation (1) [1]:

$$\log(q_e - q_t) = \log q_e - k_1 t \qquad (1)$$

where q_e (mg g^{-1}) and q_t (mg g^{-1}) are the amount of Pb(II), Cd(II), Cu(II) and Zn(II) adsorbed at equilibrium and at time t (min), respectively. k_1 (min^{-1}) is the first-order sorption kinetic constant.

The pseudo-second-order equation is as following [2]:

$$\frac{t}{q_t} = \frac{1}{k_2 q_e^2} + \frac{1}{q_e} t \qquad (2)$$

where q_e (mg g^{-1}) and q_t (mg g^{-1}) are adsorption capacities of Pb(II), Cd(II), Cu(II) and Zn(II) at equilibrium and at time t (min), respectively, and k_2 (g mg^{-1} min^{-1}) is the second-order sorption rate constant.

The Webber-Morris model is described as equation (3) [3]:

$$q_t = k_{id} t^{1/2} + C_i \qquad (3)$$

where q_t (mg g^{-1}) is the amount of adsorbed Pb(II), Cd(II), Cu(II) or Zn(II) at time t (min), k_i (mg g^{-1} $min^{-1/2}$) is the diffusion rate constant and C_i is the intercept which provides an idea of the boundary layer thickness.

Langmuir isotherm model:

The isotherm data of Pb(II), Cd(II), Cu(II) and Zn(II) sorption onto adsorbents were fitted to the Langmuir model according to the equation (4) [4]:

$$\frac{C_e}{q_e} = \frac{1}{K_L q_m} + \frac{1}{q_m} C_e \quad (4)$$

where q_e (mg g^{-1}) is the amount of adsorbed metal at equilibrium, C_e (mg L^{-1}) is the equilibrium concentration of heavy metal ions in aqueous solution, q_m (mg g^{-1}) is the maximum adsorption capacity of heavy metals on adsorbents, K_L (L mg^{-1}) is a binding constant.

Thomas model:

The Thomas model is used to predict the column breakthrough capacities. The linearized form of the Thomas model is as equation (5) [5]:

$$\ln(\frac{C_{in}}{C_{out}} - 1) = \frac{k_{Th} q_m m}{Q} - k_{Th} C_{in} t \quad (5)$$

where C_{in} and C_{out} are the influent and effluent, respectively, k_{Th} is the Thomas rate constant (ml min^{-1} mg^{-1}), q_m is the maximum adsorption capacity (mg g^{-1}), Q is the flow rate (mg min^{-1}), m is the mass of adsorbent (g), and t is the adsorption time (h).

Mn(IV) content determination procedures:

Firstly, total Mn content was measured by dissolving 0.2 g of BC@MnO_2 sample in 50 ml of 0.25 mol L^{-1} hydroxylamine hydrochloride, then the resulting Mn(II) content in the solution was diluted with water and was measured by atomic absorption spectrometry (AAS) (Perkin Elmer 2380, USA). Another 0.1 g of BC@MnO_2 sample was completely dissolved in 10 ml of 0.5 mol L^{-1} $H_2C_2O_4$ and 10 ml of 1 mol L^{-1} H_2SO_4 to reduce Mn(III) and Mn(VI) to Mn(II) at 75°C. The excess $C_2O_4^{2-}$ was measured by back-titration with a standardized $KMnO_4$ solution. According to the results of above experiments, the Mn(IV) content in BC@MnO_2 adsorbent were calculated. All

chemical analyses for each sample were performed three times and the mean was recorded.

Figure S1

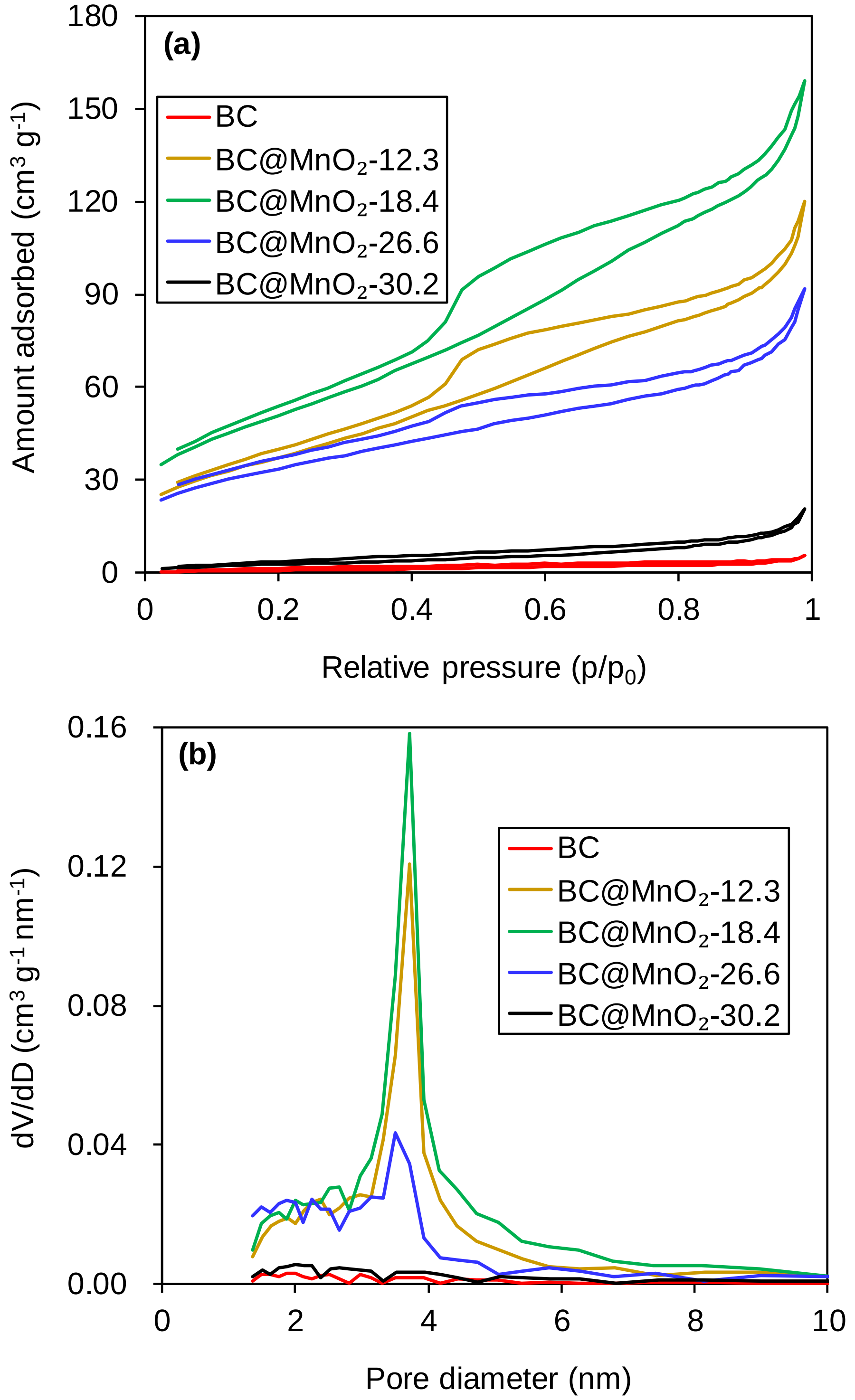

(a)
BC
BC@MnO2-12.3
BC@MnO2-18.4
BC@MnO2-26.6
BC@MnO2-30.2
Amount adsorbed (cm3 g-1)
Relative pressure (p/p0)
0
30
60
90
120
150
180
0
0.2
0.4
0.6
0.8
1
(b)
dV/dD (cm3 g-1 nm-1)
Pore diameter (nm)
0.00
0.04
0.08
0.12
0.16
2
4
6
8
10

Figure S2:

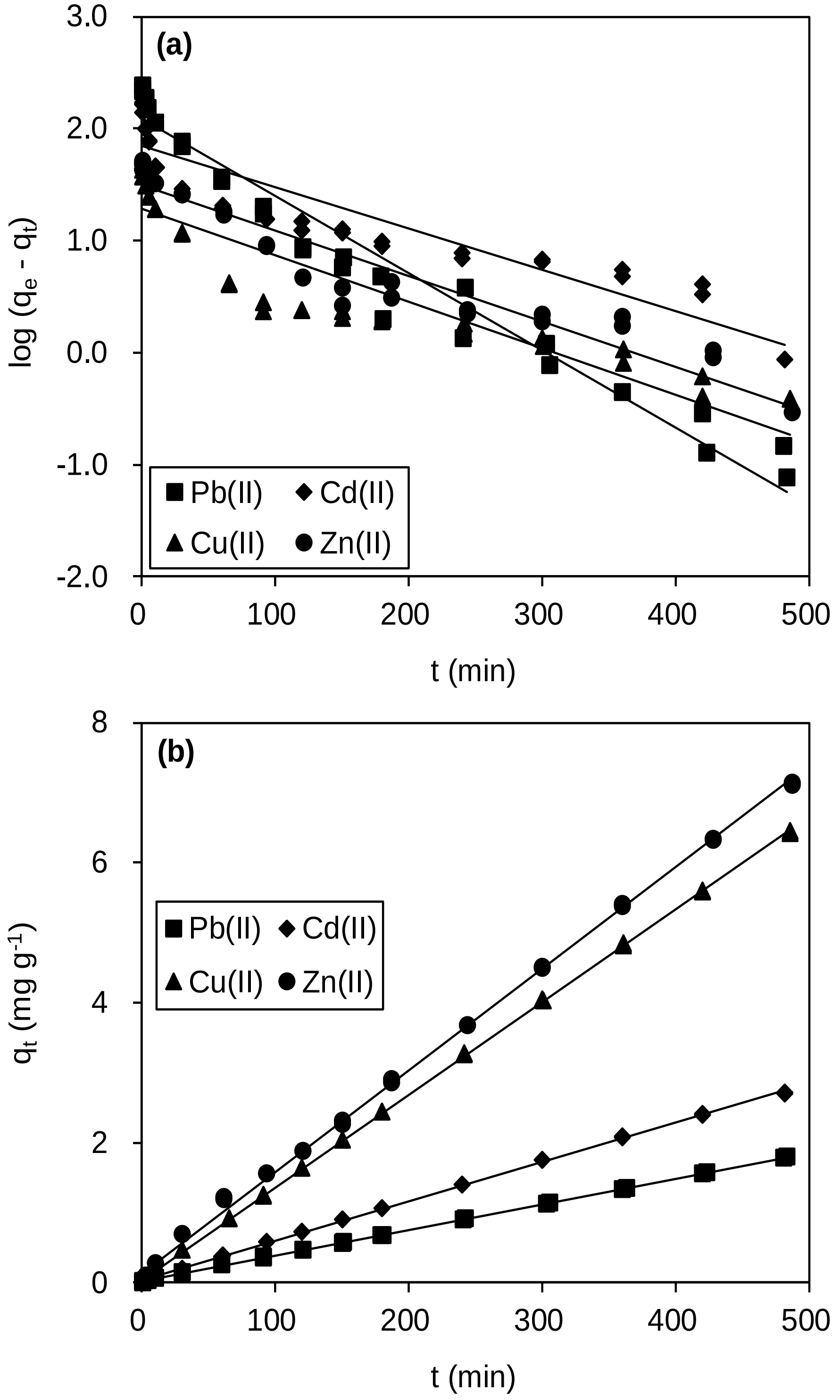

3.0
2.0
1.0
0.0
-1.0
-2.0
(a)
log (qe - qt)
Pb(II)
Cd(II)
Cu(II)
Zn(II)
0
100
200
300
400
500
t (min)
8
6
4
2
0
(b)
qt (mg g-1)
Pb(II)
Cd(II)
Cu(II)
Zn(II)
0
100
200
300
400
500
t (min)

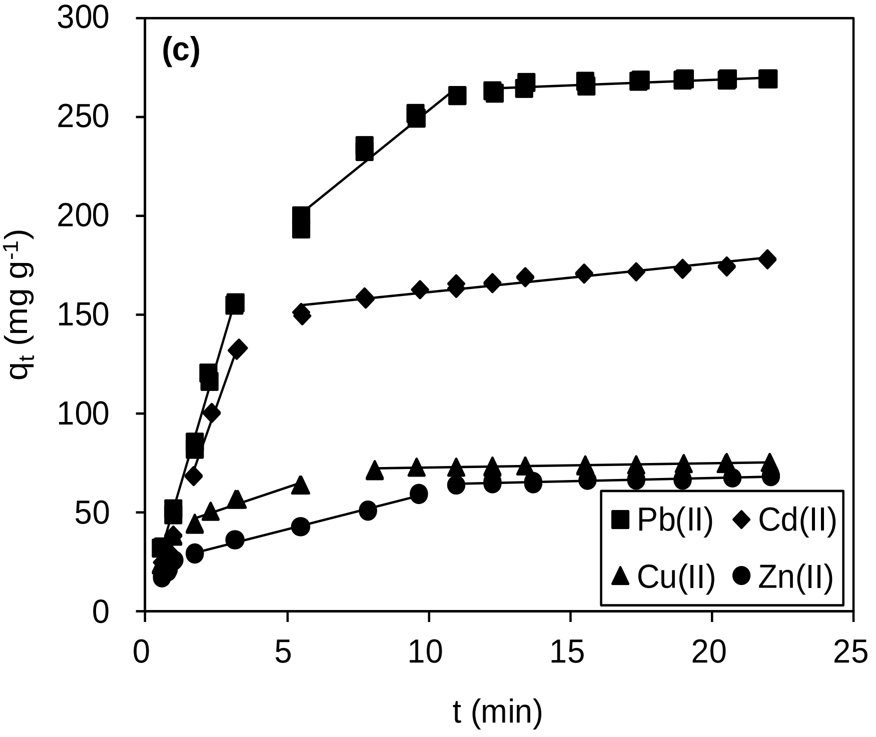
(c)
300
250
200
150
100
50
0
0
5
10
15
20
25
t (min)
q_t (mg g^-1)
Pb(II)
Cd(II)
Cu(II)
Zn(II)

Figure S3:

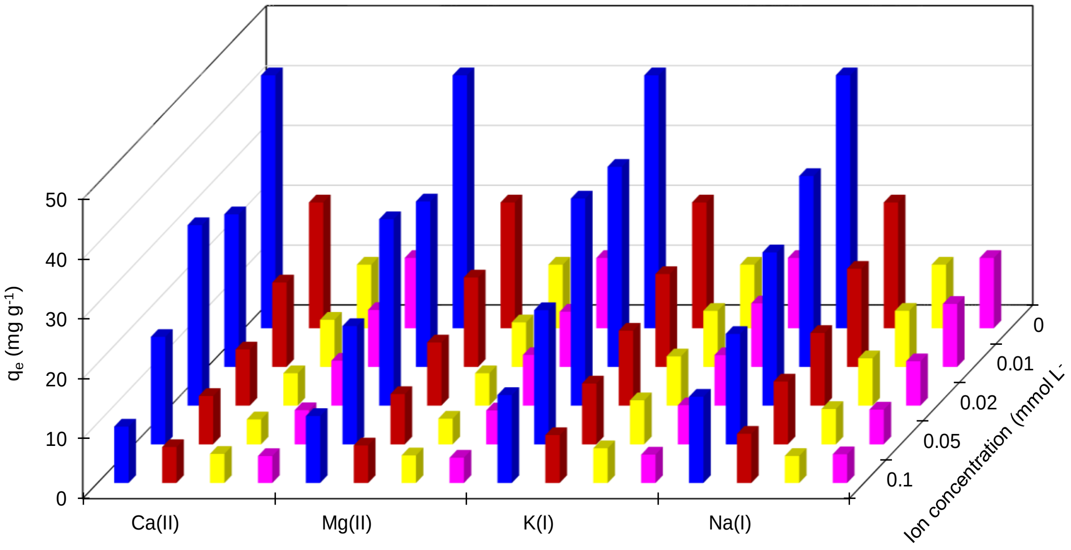

50
40
30
20
10
0
q_e (mg g^{-1})
Ca(II)
Mg(II)
K(I)
Na(I)
0
0.01
0.02
0.05
0.1
Ion concentration (mmol L-

Figure S4:

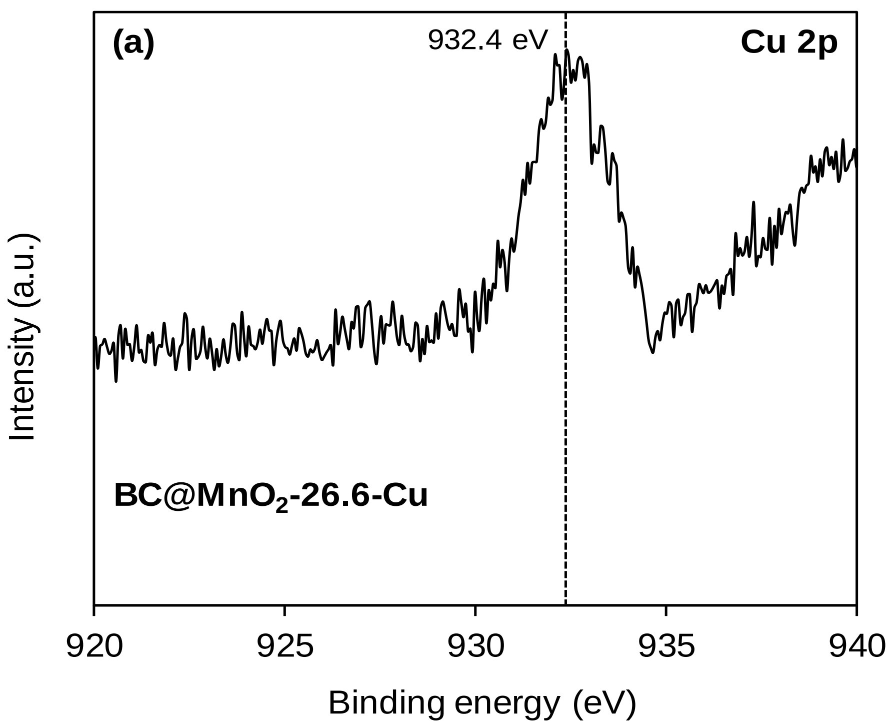

(a)
932.4 eV
Cu 2p
Intensity (a.u.)
BC@MnO2-26.6-Cu
920
925
930
935
940
Binding energy (eV)

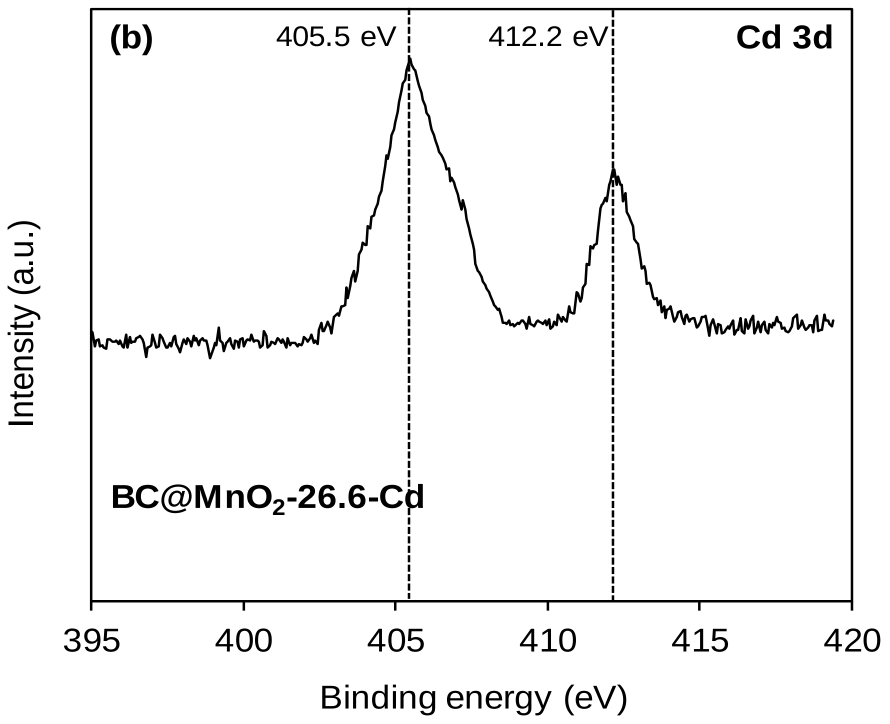

(b)
405.5 eV
412.2 eV
Cd 3d
Intensity (a.u.)
BC@MnO2-26.6-Cd
395
400
405
410
415
420
Binding energy (eV)

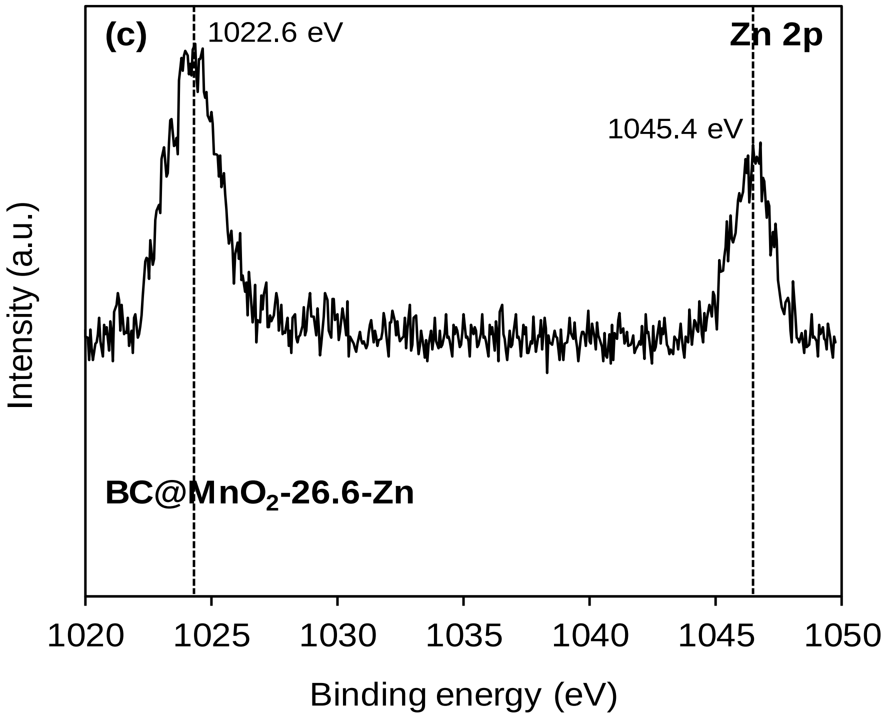

(c)
1022.6 eV
Zn 2p
1045.4 eV
Intensity (a.u.)
BC@MnO2-26.6-Zn
1020
1025
1030
1035
1040
1045
1050
Binding energy (eV)

References

[1] H.C. Trivedi, V.M. Patel, R.D. Patel. Adsorption of cellulose triacetate on calcium silicate. Eur. Polym. J. 1973, 9: 525-531.

[2] Y.S. Ho, G. McKay. Pseudo-second order model for sorption processes. Process. Biochem. 1999, 34: 451-465.

[3] W.J. Weber, J.C. Morris, Kinetics of adsorption on carbon from solution. J. Sanit. Eng. Div. Am. Soc. Civ. Eng. 89 (1963) 31-60.

[4] J.W. Tonkin, L.S. Balistrieri, J.W. Murray. Modeling sorption of divalent metal cations on hydrous manganese oxide using the diffuse double layer model. Appl. Geochem. 19 (2004) 29-53.

[5] T.Y. Liu, X. Yang, Z.L. Wang, X.X. Yan. Enhanced chitosan beads-supported Fe0-nanoparticles for removal of heavy metals from electroplating wastewater in permeable reactive barriers. Water Research, 2013, 47: 6691-6700.
